# Impact of physical activity on cardiovascular health in firefighters: Scoping review

**DOI:** 10.4102/hsag.v30i0.2713

**Published:** 2025-01-28

**Authors:** Ghaleelullah Achmat, Charlene Erasmus, Jill Kanaley, Rucia November, Lloyd Leach

**Affiliations:** 1 Department of Sport, Recreation and Exercise Science, Faculty of Community and Health Science, University of the Western Cape, Cape Town, South Africa; 2 Centre for Interdisciplinary Studies of Children, Families and Society, Faculty of Community and Health Sciences, University of the Western Cape, Cape Town, South Africa; 3 Department of Nutrition & Exercise Physiology, College of Agriculture, Food and Natural Resources, University of Missouri, Columbia, United States

**Keywords:** firefighters, health risk behaviour, physical activity, sedentary lifestyle, cardiovascular disease

## Abstract

**Background:**

Firefighters’ duties include fire response, emergency medical treatment and rescue operations. Noncompliance with physical activity (PA) guidelines increases adverse health behaviours and the risk of on-duty fatalities. While PA is known to treat cardiovascular disease (CVD), its impact on risky health behaviours in firefighters is under-researched.

**Aim:**

This scoping review aimed to evaluate PA’s effects on firefighters’ cardiovascular health.

**Method:**

The review followed PRISMA-ScR and PRISMA Protocol standards, involving a comprehensive search across databases like Cochrane, PubMed, Medline, EbscoHost, Web of Science, Academic Search Complete, CINAHL (EBSCO), SAGE Journals, ScienceDirect and Scopus, covering publications up to June 2023. The purpose was to compile evidence on PA programs’ effects on fire and rescue services (FRS).

**Results:**

Five intervention studies were included, examining PA effects on firefighters with smoking habits, poor diet, alcohol consumption and sedentary lifestyles. These interventions recommend 150 minutes per week of aerobic, flexibility and strength activities. Firefighters should be guided to initiate and maintain 150 minutes of PA weekly to promote health strategies.

**Conclusion:**

The study concludes that integrating lifestyle changes with low- and moderate-intensity PA into fire services is crucial for improving health risk behaviours (HRBs). Implementing multilevel interventions is necessary to drive policy changes supporting firefighters facing HRBs.

**Contribution:**

Educating firefighters about these behaviours is essential, fostering an understanding of healthy alternatives.

## Introduction

Firefighting is a perilous profession that exposes firefighters to significant physical and mental strain, potentially jeopardising their health and overall well-being. When firefighters engage in health risk behaviours (HRBs), they face an increased likelihood of experiencing cardiovascular (CV) incidents that could be fatal (Kales et al. 2003; Soteriades et al. 2011). The risk of CV line-of-duty death is heightened by extremely high heart rates during fire suppression activities (Kales et al. 2003; Soteriades et al. 2011). The prevalence of health issues can be attributed to high-risk behaviours that elevate the likelihood of disease or injury, ultimately leading to disability, mortality or social challenges (Kales et al. 2003; Soteriades et al. 2011). This alarming fact may be attributed to the unique circumstances of the firefighting profession that include high levels of stressful behaviour (Kales et al. 2003; Soteriades et al. 2011). Understandably, firefighters experience significant stress, as they are concerned not only for their safety but also for the safety of their colleagues and the general public, whose well-being is their primary concern (Banes 2014; Kales et al. 2003; Soteriades et al. 2011). Overexertion during strenuous duties is a major cause of line-of-duty deaths among firefighters, accounting for 47% of firefighter fatalities (Banes 2014; Kales et al. 2003; Soteriades et al. 2011). It is indeed a comforting assumption that firefighters, who are entrusted with the responsibility of taking care of others, are strong and healthy enough to effectively carry out their duties (Banes 2014; Kales et al. 2003; Soteriades et al. 2011). However, many firefighters have untreated or undiagnosed conditions such as hypertension, hyperlipidaemia and obesity, along with poor dietary habits and suboptimal physical fitness (Banes 2014; Kales et al. 2003; Soteriades et al. 2011). Common high-risk behaviours include violence, alcoholism, tobacco use disorder, risky sexual behaviours, eating disorders and a sedentary lifestyle (Banes 2014; Kales et al. 2003; Schuhmann et al. 2022; Soteriades et al. 2011). These high-risk behaviours have the ability to negatively impact on CV health. (Banes 2014; Kales et al. 2003; Soteriades et al. 2011). The development and progression of cardiovascular diseases (CVDs) are associated with a sedentary lifestyle characterised by smoking, poor nutrition, alcohol abuse and physical inactivity, often referred to as ‘SNAP’ (Banes 2014; Carey et al. 2021; Kales et al. 2003; Soteriades et al. 2011).

These risky SNAP behaviours are recognised as significant contributors to poor CV health. (Carey et al. 2011; Maloney et al. 2021). Furthermore, these high-risk behaviours are a critical health concern among firefighters, who are more susceptible to CVD events because of intermittent periods of intense physical activity (PA) while on-duty (Banes 2014; Carey et al. 2021; Kales et al. 2003; Maloney et al. 2021; Soteriades et al. 2011). Research indicates that new recruit firefighters typically exhibit higher levels of fitness and better health at the onset of their careers; however, these standards and levels of health and fitness often decline throughout their service in the fire department (Banes 2014; Carey et al. 2021; Kales et al. 2003; Maloney et al. 2021; Soteriades et al. 2011). Occupational exposure to smoke and carbon monoxide levels poses a significant hazard that amplifies CV risk among firefighters (Maloney et al. 2021; Schuhmann et al. 2022). Cultural factors such as shift work and team cohesion exert multiple levels of influence on firefighters’ decisions regarding adoption of positive health behaviours (Banes 2014; Carey et al. 2021; Kales et al. 2003; Maloney et al. 2021; Schuhmann et al. 2022; Soteriades et al. 2011). Firefighters encounter intermittent peaks of strenuous work within prolonged periods of inactivity; these extended phases of sedentary behaviour have been demonstrated to elevate the risk of CVD and other chronic illnesses (Butry et al. 2019; Latosinski et al. 2024; Schuhmann et al. 2022). Studies considering both direct and indirect costs indicate that firefighter injuries result in annual costs ranging from $1.6 billion to $5.9 billion (Butry et al. 2019; Gronek et al. 2020; Kuehl et al. 2013). Numerous studies support the conclusion that physiological overexertion and musculoskeletal disorders may be the primary sources of firefighter injuries (Butry et al. 2019; Gronek et al. 2020; Kuehl et al. 2013; Latosinski et al. 2024). Physical inactivity, in combination with multiple high-risk behaviours, significantly contributes to the accumulation of CV events (Butry et al. 2019; Latosinski et al. 2024; Schuhmann et al. 2022). Modifiable behaviours such as SNAP all elevate the risk of noncommunicable diseases (NCDs), on-duty CVD events and mortality (Banes 2014; Butry et al. 2019; Carey et al. 2011; Gronek et al. 2020; Kales et al. 2003; Kuehl et al. 2013; Latosinski et al. 2024; Maloney et al. 2021; Schuhmann et al. 2022; Soteriades et al. 2011).

In the U.S., each fire department is tasked with establishing its standards for firefighters’ fitness levels (Banes 2014; Kales et al. 2003; Soteriades et al. 2011). While the National Fire Protection Association (NFPA) in the U.S. provides established fitness standards, the adoption and enforcement of these standards is discretionary and varies across departments (Banes 2014; Kales et al. 2003; Soteriades et al. 2011). Consequently, there is considerable diversity in fitness levels among firehouses (Banes 2014; Carey et al. 2011). The absence of clear and consistent expectations for an ideal fitness standard makes it challenging for members of the fire service to determine the types of exercises that are suitable and beneficial for their short- and long-term health (Maloney et al. 2021; Schuhmann et al. 2022). In addition, the food options in surrounding communities and within firehouses often present a barrier to achieving and sustaining healthy standards (Wooding et al. 2018). Firefighters frequently report that unhealthy choices such as donuts, pizza, and other fast and unhealthy options are readily available and difficult to resist, while fresh and healthy options are scarce (Jahnke et al. 2016; Wooding et al. 2018). Furthermore, the absence of a regular routine and predictable hours may also significantly contribute to firefighters’ challenges in maintaining their health (Haddock 2011; Jahnke et al. 2016; Wooding et al. 2018). It is concerning that researchers have found a significant link between short, irregular and disrupted sleeping patterns and obesity in adults (Haddock 2011; Jahnke et al. 2016; Wooding et al. 2018). A meta-analytic review has shown that periods of sleep less than 5–7 h per night, which is common among firefighters, are associated with a higher risk of death, regardless of age, gender and socio-economic status (Cappuccio et al. 2008). Unfortunately, a cycle often develops where insufficient sleep leads to unhealthy factors, such as obesity, and obesity in turn increases the likelihood of being a short sleeper (Cappuccio et al. 2008; Frost et al. 2021). Furthermore, the unpredictable nature of a firefighter‘s duty and their sleeping patterns adds to the challenge (Cappuccio et al. 2008; Frost et al. 2021). It is conceivable that any one of these barriers alone may hinder the development of a healthy lifestyle, but firefighters often face many, if not all, of the aforementioned barriers, placing them at an even greater risk (Cappuccio et al. 2008; Frost et al. 2021; Haddock 2011). Firefighters face significant psychological stress during their work, which can result in mental and behavioural health issues that often remain unreported (Cappuccio et al. 2008; Frost et al. 2021; Haddock 2011). Jahnke and colleagues found from a sample of 332 career firefighters (CFFs) that the lack of national standards for firefighter health, departmental mandates and financial support for health and wellness were major barriers to engaging in healthy behaviours (Frost et al. 2021). To better understand this situation, Thews et al. (2020) surveyed 314 firefighters who participated in a survey, with many reporting cultural challenges stemming from the expectations set by their administration and colleagues (Thews et al. 2020). The results highlight a clear need for a shift in the culture of the fire service, advocating for a more supportive atmosphere that promotes the well-being of firefighters (Thews et al. 2020). Similarly, Gonzalez and colleagues discovered from a sample of CFFs that barriers to health and wellness included, among other factors, the demanding nature of firefighting and the stress they faced (Gonzalez et al. 2024). Additionally, participants emphasised their struggle to find healthy, affordable, and easily prepared foods while on-duty and expressed openness to improving their food choices (Gonzalez et al. 2024; Staley, Weiner & Linnan 2011). They also identified personal motivation and time limitations as crucial factors in increasing PA and living healthy lives, but were unable to offer concrete suggestions for effective interventions (Gonzalez et al. 2024; Staley et al. 2011; Thews et al. 2020). Conversely, regular exercise and PA are linked to broad health benefits and a markedly reduced risk of CVD and mortality (Banes 2014; Carey et al. 2011; Kales et al. 2003; Maloney et al. 2021; Soteriades et al. 2011). Fire department worksite health, wellness and fitness policy programmes should actively address firefighters’ CV risks (Banes 2014; Butry et al. 2019; Carey et al. 2011; Gronek et al. 2020; Kales et al. 2003; Kuehl et al. 2013; Latosinski et al. 2024; Maloney et al. 2021; Schuhmann et al. 2022; Soteriades et al. 2011).

### Aim

This scoping review aims to determine the effects of PA on the CV health of firefighters.

## Methods

### Study design

A scoping review involves a systematic and iterative process to identify and synthesise existing or emerging literature on a specific topic (Arksey & O’Malley 2005; Yassin 2020). This scoping review aimed to evaluate how HRBs and PA impact CV health in firefighters. Included reports were reviewed and evaluated according to the six-step framework developed by Arksey and O’Malley (2005), and the Preferred Reporting Items for Systematic Reviews and Meta-Analyses extension for Scoping Reviews (PRISMA-ScR) checklist; furthermore, it followed the four-phase flow diagram of Arksey and O’Malley (2005) (Yassin 2020).

### Research question

The following research question was formulated using the PEO (Population, Exposure, Outcome) method, which represented the: (1) population of interest (firefighters); (2) exposure (PA); and (3) the outcome of interest (CV health of firefighters) (Britton, Rosenwax & McNamara 2021; Yassin 2020). The research question was: *What are the effects of physical activity and health risk behaviours on cardiovascular health?*

### Inclusion and exclusion criteria

Studies that were included in the current review were required to: (1) be published from inception until June 2023; (2) have used a quantitative, qualitative or mixed methods methodology; (3) be in the English language; (4) be full-text and peer-reviewed; (5) include firefighters who have one or more HRBs; and (6) examine and report on the effects of PA on the HRBs of firefighters (Yassin 2020). For more information on the aim and objectives of this review, refer to the published protocol (Achmat et al. 2023; Malik, Blake & Suggs 2014). As a result of the paucity of current literature, studies including firefighters with SNAP HRBs were included in the current review. Conversely, studies were excluded from the current review: (1) if they were published before 2002, (2) were not in English, (3) and (4) if they failed to report on the PA and HRBs of firefighters (Achmat et al. 2023; Malik et al. 2014; Yassin 2020).

### Search strategy and selection criteria

The University of the Western Cape’s online library was utilised to access and search the following electronic databases: Cochrane database, PubMed, Medline, EBSCOhost, Web of Science, Academic Search Complete, CINAHL (EBSCO), SAGE Journals, ScienceDirect and Scopus. Searches included a combination of terms from medical subject headings (MeSH) and keywords in the title, abstract and text. All articles that qualified in terms of PECO and the eligibility criteria were used.

### Search terms

Various terms were used for ‘population’ (e.g. firefighter), ‘intervention’ (e.g. PA) and ‘outcomes’ (e.g. HRBs). Reports having biases were excluded. The following strings of search terms and keywords were entered into the respective databases: all terms were combined with ‘and’ Physical Activity, ‘or’ Exercise, ‘or’ Fitness, ‘or’ Physical Exercise ‘and’ Health Risks ‘or’ Health Risk Behaviour ‘and’ Firefighters ‘or’ Fire fighters ‘or’ Fire Service ‘or’ Firefighting (Achmat et al. 2023; Malik et al. 2014). All articles published from inception until June 2023 were searched. Grey literature, such as government reports, institutional documents, dissertations (published as peer-reviewed articles), books, book chapters, conference abstracts or proceedings, blogs, newsletters, or any opinion-based publications and commentaries, were excluded. The scoping review considered all studies utilising quantitative, qualitative and mixed methods studies (Achmat et al. 2023; Arksey & O’Malley 2005; Armstrong et al. 2011; Britton et al. 2015; Gonzalez et al. 2024; Gottlieb et al. 2021; Jørgensen, Hilden & Gøtzsche 2006; Malik et al. 2014; Salama et al. 2016; Staley et al. 2011; Yassin 2020).

### Method of review

**FIGURE 1:**
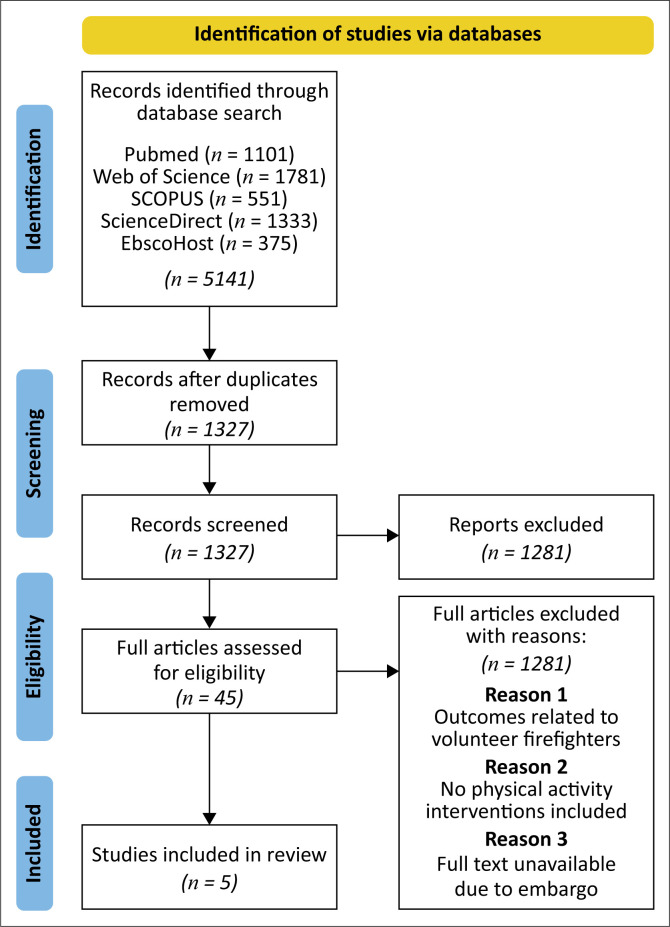
PRISMA flow diagram for the scoping review process.

The review procedure consisted of four phases to identify relevant studies for this scoping review using the search criteria previously described (Achmat et al. 2023; Arksey & O’Malley 2005; Armstrong et al. 2011; Britton et al. 2015; Gonzalez et al. 2024; Gottlieb et al. 2021; Jørgensen et al. 2006; Malik et al. 2014; Salama et al. 2016; Staley et al. 2011; Yassin 2020). The first phase included screening titles of articles; the second phase consisted of screening abstracts; the third phase identified the eligible articles; and the fourth phase reviewed the full-text articles. In addition, the reference lists of the full-text articles were retrieved to search for potentially eligible studies (Achmat et al. 2023; Malik et al. 2014; Yassin 2020). The primary researcher and two independent researchers screened the titles of prospective studies (Achmat et al. 2023; Malik et al. 2014; Yassin 2020). Pertinent full texts of the abstracts were retrieved for rigour and eligibility by two independent researchers. Regarding the scoping review PRISMA-ScR flow chart, all articles are identified at each of the four phases of the review, refer to Figure 1 (Achmat et al. 2023; Arksey & O’Malley 2005; Armstrong et al. 2011; Britton et al. 2015; Gonzalez et al. 2024; Gottlieb et al. 2021; Jørgensen et al. 2006; Malik et al. 2014; Salama et al. 2016; Staley et al. 2011; Yassin 2020). At each point, studies that did not meet the inclusion criteria were eliminated, and duplicates were manually sought and removed (Achmat et al. 2023; Malik et al. 2014; Yassin 2020). All disagreements regarding the methodological quality and inclusion of studies were discussed by a third research reviewer until consensus was reached (Achmat et al. 2023; Arksey & O’Malley 2005; Armstrong et al. 2011; Britton et al. 2015; Gonzalez et al. 2024; Gottlieb et al. 2021; Jørgensen et al. 2006; Malik et al. 2014; Salama et al. 2016; Staley et al. 2011; Yassin 2020). Studies meeting the predetermined threshold for inclusion proceeded to the level of inclusion and were subjected to the process of data extraction (Achmat et al. 2023; Arksey & O’Malley 2005; Armstrong et al. 2011; Britton et al. 2015; Gonzalez et al. 2024; Gottlieb et al. 2021; Jørgensen et al. 2006; Salama et al. 2016; Staley et al. 2011; Yassin 2020).

### Data extraction and synthesis

Data from eligible studies were recorded on the Data Extraction form (Table 1), which was followed by the current guidelines for conducting scoping reviews, recorded data included: Publication details, country of study, objective(s) of the study, study design, sample size, and a summary of findings. For synthesis, extracted information was grouped into themes based on PA interventions (Achmat et al. 2023; Arksey & O’Malley 2005; Armstrong et al. 2011; Britton et al. 2015; Gonzalez et al. 2024; Gottlieb et al. 2021; Jørgensen et al. 2006; Malik et al. 2014; Salama et al. 2016; Staley et al. 2011; Yassin 2020). These PAs reported on HRBs intervention strategies received by firefighters such as frequency, intensity, type and duration of the PA (Achmat et al. 2023; Arksey & O’Malley 2005; Armstrong et al. 2011; Britton et al. 2015; Gonzalez et al. 2024; Gottlieb et al. 2021; Jørgensen et al. 2006; Malik et al. 2014; Salama et al. 2016; Staley et al. 2011; Yassin 2020). The data extracted by two investigators sought information on the extent of the need for PAs to impact the CV health of firefighters and the service delivery of PA interventions within the fire and rescue services (FRS). All extracted data were reviewed for accuracy and correctness by a third investigator (Achmat et al. 2023; Arksey & O’Malley 2005; Armstrong et al. 2011; Britton et al. 2015; Gonzalez et al. 2024; Gottlieb et al. 2021; Jørgensen et al. 2006; Malik et al. 2014; Salama et al. 2016; Staley et al. 2011; Yassin 2020).

**TABLE 1:** Data extraction form.

No.	Authors	Sample	Geographical location	Aim	Findings
1.	Elliot et al. (2004)	*n* = 33 CFFs	U.S.	To assess the efficacy of two worksite health promotion interventions	Questionnaire responses related to knowledge domains, barriers to exercise, and beliefs about home life did not show any significant effects. However, both the team-based and one-on-one intervention firefighters experienced significant reductions in LDL cholesterol compared to the control group. The one-on-one intervention also led to a significant decrease in behaviours associated with higher dietary fat intake. Additionally, the team-based intervention resulted in a significant increase in personal exercise practices among firefighters, compared to the control group. Both the team-based and individual-oriented interventions brought about significant changes in health behaviours, potential mediating constructs, and secondary laboratory outcomes. The one-on-one approach may be more suitable for a clinical setting, as office-based health promotion interventions have limited effectiveness. On the other hand, the team strategy represents a relatively new approach for modifying adults’ health behaviours, and the study’s results suggest that it is feasible and well-received by participants.
2.	Moe et al. (2002)	*n* = 600 CFFs	U.S.	To evaluate the efficacy of two intervention strategies for improving nutrition and PA practices in fire fighters: a team-centred programme and a one-on-one format targeting the individual	Firefighters exhibit a concentration of the same detrimental behaviours and health risks that are commonly seen in the wider US population. The unique work structure of firefighters makes them well-suited for a team-centred approach to behaviour change. This approach, rooted in Social Learning Theory, emphasises a team of firefighters who work together during the same shift. If this team-centred approach proves effective, it could offer a cost-efficient means of influencing behaviour and could be implemented in fire departments and other team environments. The one-on-one intervention, on the other hand, incorporates the Transtheoretical Model of behaviour change and utilises Motivational Interviewing as its counselling strategy, and is more suitable for the typical provider-client clinic setting. The findings from PHLAME will yield insights into the process and outcomes of these models’ effectiveness in bringing about health behaviour change.
3.	MacKinnon et al. (2010)	*n* = 599 CFFs	U.S.	To describe the effects of two worksite health promotion programmes for firefighters Both immediate outcomes and the long-term consequences over 4 years following the interventions	Both the team-centred peer-taught curriculum and the individual motivational interviewing intervention showed positive impacts on BMI, with the team approach also influencing nutrition behaviour and PA after 1 year. While many differences between the intervention and control groups diminished in subsequent annual assessments, the overall trend of behaviours across time was positive for all groups, indicating lasting effects and the spread of programme benefits across experimental groups within the worksites. Despite the 1 year programme effects not persisting over time, the long-term behavioural patterns suggested that these worksites, as a whole, were healthier more than 3 years after the interventions.
4.	Poston et al. (2013)	*n* = 1002 CFFs	U.S.	To evaluate the health of firefighters from departments with well-developed health promotion programmes and≈compare them with departments not having such programmes	Firefighters in departments deliberately chosen for their robust wellness programmes in Washington were found to be healthier across various health dimensions, exhibiting better overall body composition, lower rates of obesity and anxiety disorders, reduced smoking prevalence, and higher levels of PA and/or exercise, as well as increased job satisfaction. Worksite health promotion programmes have generally shown positive effects in addressing several health issues prioritised by the US Fire Service, including body composition, PA, and tobacco and alcohol use. This research adds to the current body of evidence by showing that firefighters in departments with robust health promotion programmes that align with the goals of the WFI and those recommended by the American Heart Association for comprehensive worksite health promotion programmes were in better health and displayed greater operational readiness compared to firefighters in departments without such programmes.
5.	Ranby et al. (2011)	*n* = 397 CFFs	U.S.	To examine a health promotion intervention, improved healthy eating, and exercise behaviour among firefighters	This study highlighted areas that require attention and improvement. For instance, despite the higher PA levels among WA firefighters, the majority of firefighters in both groups did not meet the NFPA minimum post-cardiac event exercise tolerance threshold. Both groups exhibited a high prevalence of smokeless tobacco use and binge drinking/heavy drinking, with estimates consistent with data from previous population-based studies. This suggests that more focus on addressing these behavioural health issues is urgently needed in the fire service.

Note: Please see the full reference list of this article for details on the articles cited: https://doi.org/10.4102/hsag.v30i0.2713.

U.S., United States; CFF, career firefighters; PA, physical activity; No, number.

### Data extraction and analysis

Data extraction utilised a self-constructed sheet aligned with the Cochrane Data Extraction and Assessment Form guidelines (Jørgensen et al. 2006). This form was developed through the adoption and customisation of the data collection form for intervention reviews (Jørgensen et al. 2006). The following information was extracted from each study, namely author/s, publication year, aim, problem statement, target population, geographical location, study design, sampling method, sample size, data collection methods, and instruments, methods of analysis, findings, and conclusions (Achmat et al. 2023; Arksey & O’Malley 2005; Armstrong et al. 2011; Britton et al. 2015; Gonzalez et al. 2024; Gottlieb et al. 2021; Jørgensen et al. 2006; Salama et al. 2016; Staley et al. 2011; Maart, Adam & Frantz 2014; Malik et al. 2014; Yassin 2020). To reduce bias, the primary researcher piloted a data extraction sheet. The study used a meta-synthesis analysis consisting of a descriptive meta-synthesis and theory explication to critically analyse and discuss emerging themes from the findings of the included studies (Achmat et al. 2023; Arksey & O’Malley 2005; Armstrong et al. 2011; Britton et al. 2015; Gonzalez et al. 2024; Gottlieb et al. 2021; Jørgensen et al. 2006; Salama et al. 2016; Staley et al. 2011; Maart et al. 2014; Malik et al. 2014; Yassin 2020).

### Ethical considerations

Ethical permission was obtained from the University of the Western Cape’s Senate Research Ethics Committee, BM21/02/07. All studies included are published, peer-reviewed articles available in the public domain, ensuring transparency (Achmat et al. 2023; Malik et al. 2014; Yassin 2020).

## Review findings

### Process of results

The initial electronic search strategy yielded a total of 5,141 potential titles across databases. After the removal of duplications, 1326 prospective titles were screened for relevance to this study, resulting in the exclusion of 3814 titles. The remaining 1327 titles were then reviewed by an abstract for relevance and suitability, resulting in the exclusion of 1281 articles. As a result of the paucity of literature, the citation lists of the remaining 45 sources were reviewed for further identification of prospective studies; however, no new studies were identified. The main reason for exclusion was because the outcomes were volunteer firefighters (*n* = 16), no PA intervention was included (*n* = 21) and the full-text was not available because of an embargo (*n* = 3). A total of five studies were thus included in the current review and underwent data extraction. A visual representation of the screening process at each level of review is presented in Figure 1 (Arksey & O’Malley 2005).

### Summary of studies

This scoping review included five intervention-based studies with diverse methodologies. The study populations consisted of CFFs affected by HRBs, and the settings of the studies varied geographically across the United States of America (5 studies) (MacKinnon et al. 2010; Moe et al. 2002; Ng et al. 2021; Poston et al. 2013; Ranby et al. 2011). The sample sizes ranged from 28 to 1002 individuals, and all five studies focused on interventions aimed at investigating the effects of PA on firefighters with HRBs (MacKinnon et al. 2010; Moe et al. 2002; Ng et al. 2021; Poston et al. 2013; Ranby et al. 2011). Physical activity was identified as the main variable associated with the poor health behaviours of firefighters extensively discussed in relation to their well-being (MacKinnon et al. 2010; Moe et al. 2002; Ng et al. 2021; Poston et al. 2013; Ranby et al. 2011). The review indicates that firefighters face significant challenges related to HRBs and PA on CV health, with more than half of on-duty heart attacks and deaths being linked to CVD. Despite this, most firefighters do not adhere to exercise and dietary recommendations (MacKinnon et al. 2010; Moe et al. 2002; Ng et al. 2021; Poston et al. 2013; Ranby et al. 2011). Studies reported that, sedentary lifestyles, type 2 diabetes, obesity, hypertension, dyslipidaemia and chronic musculoskeletal complaints are prevalent among firefighters, posing a greater risk of injury, absenteeism, disability and higher healthcare costs (MacKinnon et al. 2010; Moe et al. 2002; Ng et al. 2021; Poston et al. 2013; Ranby et al. 2011). Given the demanding nature of the firefighting occupation, intervention strategies targeting HRBs aim to improve firefighters’ quality of life within both individual and group settings (MacKinnon et al. 2010; Moe et al. 2002; Ng et al. 2021; Poston et al. 2013; Ranby et al. 2011). These strategies focus on health promotion behaviours targeting specific nutrition and PA practices among firefighters, with motivational interviewing being utilised as a tool to modify behaviour and promote healthier habits (MacKinnon et al. 2010; Moe et al. 2002; Ng et al. 2021; Poston et al. 2013; Ranby et al. 2011).

### Multiple health risk behaviours and physical activity among firefighters

This scoping review highlights several challenges and barriers faced by firefighters in adopting healthy behaviours and reducing HRBs (MacKinnon et al. 2010; Moe et al. 2002; Ng et al. 2021; Poston et al. 2013; Ranby et al. 2011). These include smoking, unhealthy nutritional habits, alcohol consumption, sedentary periods, long working hours, winter weather, lack of access to equipment, lack of motivation, unfamiliarity with exercise training, fear of fatigue or injury, and time constraints for food preparation (MacKinnon et al. 2010; Moe et al. 2002; Ng et al. 2021; Poston et al. 2013; Ranby et al. 2011). Limited knowledge of HRBs and health attitudes within the firefighting community, as well as negative social norms surrounding outdoor activity, further contribute to these challenges (MacKinnon et al. 2010; Moe et al. 2002; Ng et al. 2021; Poston et al. 2013; Ranby et al. 2011). Further research confirms that insufficient PA can result in social issues, lifestyle-related chronic diseases, disability, and ultimately death because of numerous HRBs (Amodeo & Nickelson 2020). Amodeo and Nickelson (2020) reported that firefighters did not perceive themselves as high-risk for CVD despite a culture of stress, cigarette smoking, quick and easy foods, unhealthy nutritional intake, alcohol consumption and not meeting recommended PA guidelines (Strait 2021). Failing to meet the recommended levels of PA increases the risk of heart disease and cardiac incidents during emergency calls for firefighters (Elliot et al. 2007; Ng et al. 2021; Poston et al. 2013; Ranby et al. 2011). A systematic review by Strait 2021 highlighted firefighters’ poor PA and fitness levels, dietary habits and work food environment contributing to higher CVD prevalence (Strait 2021). Despite firefighters experiencing significantly higher levels of CV events than other emergency rescue services, less than the recommended 150 min of PA per week is accumulated (Elliot et al. 2007; Ng et al. 2021; Poston et al. 2013; Ranby et al. 2011; Rhea, Alvar & Grey 2004; Strait 2021). Firefighting involves prolonged on-duty periods of sedentary activity, which can be particularly risky for firefighters due to the sudden and rapid increase in heart rate that occurs when the fire alarm bell sounds (Banes 2014; Carey et al. 2011; Kales et al. 2003; Maloney et al. 2021; Schuhmann et al. 2022; Soteriades et al. 2011). This underscores the importance of regular PA in the firefighting profession, as it directly impacts occupational tasks and overall health (Banes 2014; Carey et al. 2011; Kales et al. 2003; Maloney et al. 2021; Schuhmann et al. 2022; Soteriades et al. 2011). This sympathetic physiological response can compound the risk of heart attacks for firefighters, making it important for them to maintain good CV health and manage their risk factors (Banes 2014; Carey et al. 2011; Kales et al. 2003; Latosinski et al. 2024; Maloney et al. 2021; Schuhmann et al. 2022; Soteriades et al. 2011). The present results suggest that cardiorespiratory fitness is the most significant factor in achieving optimal performance among firefighters (Banes 2014; Carey et al. 2011; Maloney et al. 2021). Research showed that new firefighter recruits have better fitness levels and maximum oxygen uptake compared to older, experienced firefighters, with lower cardiorespiratory fitness correlating with decreased occupational performance as firefighters age and accumulate more years of service (Banes 2014; Carey et al. 2011; Kales et al. 2003; Latosinski et al. 2024; Maloney et al. 2021; Schuhmann et al. 2022; Soteriades et al. 2011). This finding underscores the importance of maintaining good CV health for firefighters, especially as they age in the fire service (Butry et al. 2019; Kuehl et al. 2013; Latosinski et al. 2024). It implies that firefighters with better cardiorespiratory fitness are likely to perform their occupational tasks more effectively compared to those with lower levels of fitness (Butry et al. 2019; Kuehl et al. 2013; Latosinski et al. 2024). Therefore, it is crucial for older firefighters to prioritise activities and exercise that improve their cardiorespiratory fitness (Butry et al. 2019; Gronek et al. 2020; Kuehl et al. 2013). Regular aerobic exercises such as running, cycling or swimming can be beneficial for maintaining and improving CV health (Banes 2014; Butry et al. 2019; Carey et al. 2011; Gronek et al. 2020; Haddock 2011; Jahnke et al. 2016; Kuehl et al. 2013; Latosinski et al. 2024; Maloney et al. 2021; Soteriades et al. 2011; Schuhmann et al. 2022; Wooding et al. 2018). In addition, following a healthy lifestyle that includes a balanced diet and avoiding tobacco use can also contribute to optimal cardiorespiratory fitness (Haddock 2011; Jahnke et al. 2016; Wooding et al. 2018).

### The impact of physical activities in the fire service

Poston et al. (2013) compared the health of firefighters in departments with and without health programmes by addressing body composition, fitness and behavioural health. Results showed that firefighters in wellness approach (WA) departments had lower obesity rates, met endurance standards, and had higher estimated VO2max (Poston 2013). Wellness approach firefighters were less likely to smoke or have anxiety disorders, and had higher job satisfaction (Poston 2013). However, they were more likely to report injuries to Workers’ Compensation (Armstrong et al. 2011; Elliot et al. 2004; Moe et al. 2002). It is important to notice that while cardiorespiratory fitness was found to be the most significant factor, other fitness components such as muscular endurance and strength still play a role in specific firefighting tasks (Armstrong et al. 2011; Elliot et al. 2004; MacKinnon et al. 2010; Moe et al. 2002). Therefore, a well-rounded fitness routine that incorporates both CV and strength training exercises is ideal for maintaining overall fitness and performance as a firefighter (Armstrong et al. 2011; Elliot et al. 2004; MacKinnon et al. 2010; Moe et al. 2002; Ng et al. 2021; Poston et al. 2013; Ranby et al. 2011). Collaboration among universities, governments, community members and stakeholders is crucial in providing the necessary support, infrastructure, training facilities and access to health promotion programmes, healthy eating guidelines and lifestyle modifications for firefighters (Parpa & Michaelides 2024; Poudevigne et al. 2021). Research has shown that experiential learning projects led by exercise science undergraduate students observed changes following a 10-week high-intensity functional training (HIFT) programme (Parpa & Michaelides 2024; Poudevigne et al. 2021). The professional firefighters (PFFs) trained two to three times per week during their work shifts at a vigorous intensity for 40 min (Poudevigne et al. 2021). Their resting diastolic blood pressure and resting heart rate decreased significantly (Poudevigne et al. 2021). Improvements in agility, muscular strength and readiness for change were observed by collaborating with key stakeholders and were found to be feasible and beneficial, leading to enhanced health and physical fitness for the fire services using limited resources (Parpa & Michaelides 2024; Poudevigne et al. 2021).

To address these challenges and mitigate HRBs, it is recommended that firefighters receive support and education programmes, as well as access to certified trained professionals who can implement PA interventions following the guidelines set by organisations such as the American College of Sports Medicine (ed. ACSM 2013; Moore et al. 2016). The ACSM guidelines emphasise the need for information, programmes and resources to improve nutrition and PA among firefighters to reduce CVD risk (ACSM 2013; Moore et al. 2016). These efforts aim to improve the overall health and well-being of firefighters and reduce the prevalence of HRBs within the firefighting community (Moore et al. 2016; Parpa & Michaelides 2024; Poudevigne et al. 2021). Regular PA is crucial for maintaining CV health, especially among older firefighters (Parpa & Michaelides 2024; Poudevigne et al. 2021; Moore et al. 2016). Research has shown that older firefighters, especially those aged 45 years or older, tend to become less physically active as they age (Moore et al. 2016; Parpa & Michaelides 2024; Poudevigne et al. 2021). However, it is important for them to engage in regular PA to maintain their work performance at acceptable standards (Parpa & Michaelides 2024; Poudevigne et al. 2021; Moore et al. 2016; Strait 2021; Rhea et al. 2004). Similar studies found a significant positive correlation between age and stair climb performance among firefighters (Amodeo & Nickelson 2020; Elliot et al. 2021; Moore et al. 2016; Ng et al. 2021; Parpa & Michaelides 2024; Poudevigne et al. 2021; Ranby et al. 2011; Strait 2021). Older firefighters performed significantly worse compared to younger firefighters in this task (Amodeo & Nickelson 2020; Elliot., 2021; Moore et al. 2016; Ng et al. 2021; Ranby et al. 2011; Poudevigne et al. 2021; Ranby et al. 2011; Rhea et al. 2004; Strait 2021). This correlation was particularly strong when occupational performance simulations included five or more sequential tasks (Amodeo & Nickelson 2020; Elliot., 2021; Parpa & Michaelides 2024; Poston et al. 2013; Poudevigne et al. 2021; MacKinnon et al. 2010; Moe et al. 2002; Moore et al. 2016; Ng et al. 2021; Ranby et al. 2011; Rhea et al. 2004; Strait 2021). On the other hand, age did not correlate with performance in tasks such as hose drag, victim rescue and forcible entry (Ras et al. 2024). Additionally, age significantly affected abdominal strength, relative power, push-up and sit-up repetitions performed within a minute, thus supporting earlier research indicating an age-associated decrement in physical fitness parameters among firefighters (Ras et al. 2024). The effects of ageing were found to have a larger impact on cardiorespiratory fitness, which may explain why older firefighters performed worse on the stair climb (Ras et al. 2024). It was found in the studies that older firefighters should prioritise regular PA to maintain their CV health and work performance (Ras et al. 2024; Games, Winkelmann & Eberman 2020; Heimburg et al. 2013). While ageing may affect cardiorespiratory fitness, muscular endurance and strength are also crucial for success in certain firefighting tasks (Ras et al. 2024; Games et al. 2020; Heimburg et al. 2013). Participating in cost-effective PA initiatives may help reduce HRBs and injury rates for public service workers, enabling firefighters to better meet the demands of their occupations (Ras et al. 2024; Games et al. 2020; Heimburg et al. 2013; Hershey et al. 2023).

## Discussion

This scoping review established that HRB and PA impact factors such as CV health which in turn put firefighters at risk for injury and death, thus impacting the occupational demands of firefighters. Several domains of HRBs that compound CVD were identified, namely: smoking, dietary habits, alcohol consumption and PA levels (SNAP). Marginal PA levels have contributed to firefighters’ CVD with undiagnosed or undertreated hypertension, hyperlipidaemia, obesity, alcohol consumption, cigarette smoking, as well as poor dietary habits (MacKinnon et al. 2010; Moe et al. 2002; Ng et al. 2021; Poston et al. 2013; Ranby et al. 2011). Furthermore, because of increased obesity rates, on-duty cardiac events and job stress among firefighters are well documented (MacKinnon et al. 2010; Moe et al. 2002; Ng et al. 2021; Poston et al. 2013; Ranby et al. 2011). The occurrence of CV events while firefighters are on-duty can directly impact public safety, making it a matter of global concern (MacKinnon et al. 2010; Moe et al. 2002; Ng et al. 2021; Poston et al. 2013; Ranby et al. 2011). Experiences of harmful behaviours, like overt alcohol consumption and cigarette smoking, are related to feelings of denial, while ambivalence and resistance may be stronger for addictive behaviours than non-addictive behaviours, such as fruit and vegetable consumption (Rachele, Heesch & Washington 2014; Sotos-Prieto et al. 2017). Therefore, the behaviour modification process may potentially be more challenging as firefighters are required to overcome psychological and physiological resistance, which potentially requires a systematic model to develop change (Heimburg et al. 2013; Hershey et al. 2023; Rachele et al. 2014; Sotos-Prieto et al. 2017).

### Efficacy of interventions

The effectiveness of a team-based curriculum and individual counsellor meetings interventions was found to be feasible and acceptable, leading to significant reductions in weight, blood glucose levels, LDL cholesterol, systolic and diastolic blood pressure (MacKinnon et al. 2010; Moe et al. 2002; Ng et al. 2021; Poston et al. 2013; Ranby et al. 2011). These health promotion approaches also found effectiveness with individual training sessions and showed reduction in heart rate, blood pressure, dyslipidaemia, blood glucose and body weight (MacKinnon et al. 2010; Moe et al. 2002; Ng et al. 2021; Poston et al. 2013; Ranby et al. 2011). Studies reveal that firefighters have reported the culture of a team approach to PA as favourable because they receive support from extended family members and the fire service community (Mozaffarian et al. 2012; Pirlott et al. 2012; Rachele et al. 2014; Sotos-Prieto et al. 2017). The team approach enhanced coworker cohesion, personal exercise habits, and overall healthy behaviours among colleagues, while the one-on-one strategy significantly increased dietary self-monitoring, decreased fat intake, and alleviated feelings of depression (Mozaffarian et al. 2012; Pirlott et al. 2012). These findings are consistent with previous studies, in order to manage the demands of PA and lifestyle modification, motivation tools such as peer support, MI, progress monitoring, and extrinsic incentives are effective (Banes 2014; Carey et al. 2011; Kales et al. 2003; Latosinski et al. 2024; Maloney et al. 2021; Schuhmann et al. 2022; Soteriades et al. 2011). However, the team intervention did not significantly impact exercise habits or VO2 max, although they were related to the targeted mediators (Elliot et al. 2004; Latosinski et al. 2024; Maloney et al. 2021; Olofsson 2013; Schuhmann et al. 2022). These findings demonstrate the importance of deconstructing the processes of an effective programme to understand the underlying factors that drive behaviour change and refine interventions (Elliot et al. 2004; Latosinski et al. 2024; Maloney et al. 2021; Olofsson 2013; Schuhmann et al. 2022). However, these tools can help to enhance motivation and promote sustainable behaviour change among firefighters (Elliot et al. 2004; Latosinski et al. 2024; Maloney et al. 2021; Olofsson 2013; Schuhmann et al. 2022).

### Physical activity recommendations

This research revealed connections between health-related behaviours and PA, which can positively affect CV well-being and work performance. According to the ACSM, individuals should engage in either 150 min of moderate-intensity aerobic exercise or 75 min of vigorous aerobic activity per week. (ACSM 2013; Moore et al. 2016). Literature suggests these PAs should focus on improving firefighters’ aerobic capacity, body fat percentage, muscular endurance, strength and muscular power (ACSM 2013; Banes 2014; Carey et al. 2011; Kales et al. 2003; Latosinski et al. 2024; Maloney et al. 2021; Moore et al. 2016; Schuhmann et al. 2022; Soteriades et al. 2011). Annual follow-up measurements showed that the team-centred peer-taught curriculum and the individual motivational interviewing intervention positively affected BMI (Armstrong et al. 2011; Olofsson 2013). Additionally, the team-centred intervention had positive effects on nutrition behaviour and PA (Butry et al. 2019; Kuehl et al. 2013; Moe et al. 2022; Ranby et al. 2011). However, most of the differences between the intervention and control groups diminished in later annual assessments (Butry et al. 2019; Kuehl et al. 2013; Moe et al. 2022; Ranby et al. 2011). Nevertheless, the overall trajectory of behaviours over time showed positive changes for all groups, indicating lasting effects and the diffusion of programme benefits across the experimental groups within the worksites (Heimburg et al. 2013; Hershey et al. 2023; Sotos-Prieto et al. 2017). The team-based approach notably increased coworker cohesion, personal exercise habits and overall healthy behaviours, while the individual counselling strategy led to increased dietary self-monitoring, decreased fat intake and reduced feelings of depression (Butry et al. 2019; Kuehl et al. 2013; Moe et al. 2022; Ng et al. 2021; Ranby et al. 2011). The PHLAME study supports the effectiveness of team-centred and one-on-one intervention strategies in improving nutrition and PA among firefighters using Social Learning Theory and the Transtheoretical Model with Motivational Interviewing (Butry et al. 2019; Kuehl et al. 2013; Moe et al. 2022; Ng et al. 2021; Ranby et al. 2011).

### Challenges to health risk behaviour change

A significant relationship between PA and modifying behaviour change continues to exist. With the prescription of PAs and through the application of the TTM, sustainable intervention programmes can be implemented in the FRS (Nigg et al. 2011; Pennington 2021). Contrary to that, several variables negatively affecting health were also recognised by firefighters such as time limitations, expenses, social backing, consistency, self-belief, drive for longevity, disease prevention, lack of knowledge, appearance concerns, fear of gym injury, fear of fatigue during fire combat, fear of pain, societal norms, family criticism and supportive programme staff while cultural and religious aspects were also noted (Nigg et al. 2011; Patterson et al. 2013). After obtaining medical clearance for PAs, a supervised exercise programme should be implemented and consistently maintained (Banes 2014; Carey et al. 2011; Kales et al. 2003; Maloney et al. 2021; Soteriades et al. 2011). The CDD4 emphasises light-intensity PA for individuals with a chronic condition, aiming for 150 min of such PA (ACSM 2013; Moore et al. 2016). While the preferred recommendation is 150 min of moderate-intensity PA, in cases where moderate-intensity activities pose challenges for firefighters with a chronic condition, they may be substituted for light-intensity PA (ACSM 2013; Moore et al. 2016). The objective of the CDD4 is to perform activities of daily living with the goal of patients moving independently (ACSM 2013; Moore et al. 2016). These studies indicate long-term patterns of behaviours suggesting that the worksites are healthier several years after the interventions (Banes 2014; Carey et al. 2011; Kales et al. 2003; Latosinski et al. 2024; Maloney et al. 2021; Pedersen 2019; Schuhmann et al. 2022; Soteriades et al. 2011; Strauss et al. 2021). Similar reports on body composition, fitness and behavioural health of firefighters from departments with well-developed health promotion programmes compared to those without showed that firefighters in departments with wellness programmes were healthier than those in standard departments and had a lower prevalence of obesity, higher levels of endurance capacity for firefighting and higher estimated VO2max (Banes 2014; Butry et al. 2019; Carey et al. 2011; Kales et al. 2003; Kuehl et al. 2013; Latosinski et al. 2024; Maloney et al. 2021; Schuhmann et al. 2022; Soteriades et al. 2011).

Since the study examines PA in firefighters alongside other HRBs, it is challenging to determine whether poor CV outcomes stem primarily from insufficient PA or from other HRBs. Consequently, future research should isolate these SNAP variables and account for the impact of individual factors.

### Metabolic demands of fighting fire with health risk behaviours

The physical and metabolic reactions of firefighters during simulated fire fighting activities suggest that firefighters should have a minimum aerobic capacity between 33.9 mL/kg/min and 45 mL/kg/min, as determined by maximum oxygen consumption, to effectively carry out their duties safely (Banes 2014; Kales et al. 2003; Poston et al. 2013; Ranby et al. 2011; Soteriades et al. 2011). The NFPA recommends that firefighters should have a minimum aerobic capacity of 42 mL/kg/min to effectively perform their duties during firefighting tasks (Banes 2014; Kales et al. 2003; Poston et al. 2013; Ranby et al. 2011; Soteriades et al. 2011). Firefighters that engage in PA are less likely to smoke or have been diagnosed with an anxiety disorder, and they reported higher job satisfaction. However, firefighters in wellness programme departments were somewhat more likely to have reported an injury requiring Workers’ Compensation (Elliot et al. 2004; MacKinnon et al. 2010; Moe et al. 2002; Poston et al. 2013). Furthermore, firefighters with high rates of smoking cigarettes, poor nutrition and binge drinking and alcohol consumption indicated the need for more attention to these behavioural health issues in the fire service (Elliot et al. 2004; MacKinnon et al. 2010; Moe et al. 2002; Poston et al. 2013). Overall, studies suggest that well-developed health promotion programmes can have positive effects on firefighter wellness and operational readiness (Armstrong et al. 2011; Elliot et al. 2004; MacKinnon et al. 2010; Moe et al. 2002; Poston et al. 2013; Ranby et al. 2011). However, there are still areas that require greater attention, particularly problematic alcohol consumption and tobacco use (Amodeo & Nickelson 2020; Elliot et al. 2007; Ng et al. 2021). The association between healthy diet behaviour and obesity was supported in a recent cross-sectional study but not in a prospective study (Strauss et al. 2021). However, the ability to perform necessary job tasks such as pulling a victim from a burning house requires energy from food sources during job-related tasks (Banes 2014; Carey et al. 2011; Kales et al. 2003; Latosinski et al. 2024; Maloney et al. 2021; Moore et al. 2016; Schuhmann et al. 2022; Soteriades et al. 2011).

### Engaging with key stakeholders

Given the importance of the Fire Service Policy, White Paper on Fire Services, and strategic planning opportunities, their collective integration can facilitate interventions, foster awareness campaigns, and provide education to firefighters regarding HRBs and the associated risks of CVD (Ngoepe-Ntsoane 2022; Soteriades et al. 2011). Several studies have reported that the FRS implemented a range of activity intervention strategies for firefighters grappling with HRBs; therefore, one should be mindful of the resources available among key stakeholders, government, communities, neighbourhood watch, schools and universities as these aspects of the ecosystem develop in positive health behaviour change and influence firefighters well-being (Banes 2014; Carey et al. 2011; Kales et al. 2003; Maloney et al. 2021; Poudevigne et al. 2021; Schuhmann et al. 2022; Soteriades et al. 2011; Thews et al. 2020). Utilising these resources, an integrated PA model can be implemented for the FRS that involves the adoption of healthy behaviours, resulting in notable changes to the physiological and psychological well-being of the firefighters (Banes 2014; Carey et al. 2011; Kales et al. 2003; Maloney et al. 2021; Poudevigne et al. 2021; Schuhmann et al. 2022; Soteriades et al. 2011; Thews et al. 2020). Evidence suggests that exercise acts as medicine, and when the workplace accommodates firefighters by providing increased social support, addressing training equipment needs and fostering the psychosocial well-being of firefighters, it contributes to the readiness of firefighters to embrace change (Banes 2014; Carey et al. 2011; Kales et al. 2003; Maloney et al. 2021; Poudevigne et al. 2021; Schuhmann et al. 2022; Soteriades et al. 2011; Thews et al. 2020). Physical activity strategies that included reflective listening, observations, and exploring ambivalence provided sustainability and maintenance to the behaviour changes (Banes 2014; Carey et al. 2011; Kales et al. 2003; Maloney et al. 2021; Poudevigne et al. 2021; Schuhmann et al. 2022; Soteriades et al. 2011; Thews et al. 2020).

### Job performance tasks

Occupational job task performances are attained through engagement in muscle-endurance, muscle-strengthening, high-level aerobic and anaerobic power activities. These activities involve all major muscle groups at least twice a week, promoting firefighter physical activities to enhance overall fitness and occupational performance (Banes 2014; Butry et al. 2019; Carey et al. 2011; Gronek et al. 2020; Kales et al. 2003; Kuehl et al. 2013; Latosinski et al. 2024; Maloney et al. 2021; Poudevigne et al. 2021; Schuhmann et al. 2022; Soteriades et al. 2011; Thews et al. 2020). As medical costs continue to rise because of work-related illnesses, injuries, and an increase in HRB claims, there is a need for policy change in the FRS (Haddock 2011; Jahnke et al. 2016; Wooding et al. 2018). Team-based, peer-led wellness programmes have shown to be an effective feasible and cost-effective way to implement PA change in order to reduce firefighter injury and illness rates (MacKinnon et al. 2010; Moe et al. 2002; Mozaffarian et al. 2012). By implementing the 12-session peer-led health promotion programme, fire departments participating in the PHLAME TEAM programme demonstrated a positive return on investment (ROI) (Elliot et al. 2004; Kuehl et al. 2013; Moe et al. 2002; Ranby et al. 2011). This shows that if exercise prescriptions are mandated between fire stations, this may allow for a decrease in firefighter worker compensation (WC) claims (Elliot et al. 2007; MacKinnon et al. 2010; Moe et al. 2002). Further studies report that training CFFs two to three times a week during their work shifts at low intensities for 20–40 min resulted in a reduced resting diastolic blood pressure, improved impaired fasting glucose, decreased waist circumference and significantly decreased resting heart rate (Haddock 2011; Jahnke et al. 2016; Wooding et al. 2018). In addition, improvements in cardiorespiratory endurance, agility, muscular strength, the performance of firefighting tasks and physical fitness were also observed (Carey et al. 2011; Parpa & Michaelides 2024; Poston et al. 2013; Rhea et al. 2004; Staley et al. 2011). These PA interventions demonstrate that with limited resources, feasible and sustainable collaborative initiatives could develop healthy habits in the fire service with beneficial inter-professional collaboration and theory-based intervention strategies available for the public health sector (Carey et al. 2011; Parpa & Michaelides 2024; Poston et al. 2013). In an attempt to mitigate HRBs among firefighters and enhance PAs to better address occupational demands, regular medical exams, health screenings, early detection, and PA interventions have been introduced (Butry et al. 2019; Gronek et al. 2020; Kuehl et al. 2013; Wooding et al. 2018). These initiatives aim to contribute to improved treatment outcomes and enhance the overall quality of life (QOL) (Butry et al. 2019; Gronek et al. 2020; Kuehl et al. 2013; Wooding et al. 2018).

In conclusion, firefighters risk their lives to protect the property of citizens, the lives of the nation’s civilians, and the strategic and productive assets that sustain the economy of the country (Kales et al. 2003; Soteriades et al. 2011). To implement healthy behaviour change in the FRS, key stakeholders and policymakers must enhance preventative strategies that promote health policy change aligned with personal and cultural change in the FRS (Butry et al. 2019; Kuehl et al. 2013; Latosinski et al. 2024; Maloney et al. 2021; Schuhmann et al. 2022). Regular screening for HRBs, coupled with the provision of necessary training equipment and facilities, plays a critical role in creating a sustainable environment when implementing low-moderate PA programmes for firefighters with HRBs (Haddock 2011; Jahnke et al. 2016; Wooding et al. 2018).

## Conclusion

To mitigate the risk of HRBs within the FRS, policymakers are required to engage with firefighters at all levels to develop PA guidelines. The guidelines should prioritise the promotion of low-moderate PAs and the prevention of lifestyle-related diseases to benefit firefighters and promote public safety.
